# From Gut to Systemic Circulation: Molecular Strategies of Botulinum Neurotoxin Complexes

**DOI:** 10.3390/toxins18030116

**Published:** 2026-02-24

**Authors:** Juliette Mondy, Emmanuel Lemichez

**Affiliations:** Département de Microbiologie, Unité des Toxines Bactériennes, Institut Pasteur, CNRS UMR6047, INSERM U1306, Université Paris Cité, Paris 75015, France

**Keywords:** botulism, *Clostridium botulinum*, botulinum neurotoxin, BoNT, NTNH, hemagglutinin, OrfX, P47, TULIP, mucin

## Abstract

Botulinum neurotoxins (BoNTs), among the most potent biological toxins, rely on co-produced nontoxic proteins to survive harsh gastrointestinal conditions and achieve efficient systemic dissemination after oral exposure. Recent structural and functional studies have revealed how BoNTs bind to the nontoxic non-hemagglutinin (NTNH) factors to engage in interactions with either OrfXs/P47 or hemagglutinins (HAs) components for systemic dissemination. This review synthesizes recent findings that elucidate the molecular basis of NTNH-specific anchoring to the HA70 triskelion-like element or to the host protease-activated form of OrfX2, thereby highlighting divergent pathways that enhance oral toxicity. We also discuss current perspectives on the molecular mechanisms through which BoNTs, in cooperation with associated nontoxic proteins, are absorbed from the intestine.

## 1. Introduction

Foodborne botulism occurs when food contaminated with botulinum neurotoxins (BoNTs) is ingested. This contamination happens during storage under conditions that allow bacterial growth together with BoNT production [1,2,3]. Early symptoms include fatigue, blurred or double vision, dry mouth, ptosis, and difficulty speaking or swallowing [1,4]. Gastrointestinal signs such as constipation may follow. The extent of pathophysiological disorders varies significantly depending on the dose of toxin and route of exposure, with oral uptake being far less efficient than parenteral administration [4]. The lethal amount of botulinum neurotoxin A for a 70 kg human can be estimated from primate studies, with values that vary depending on the route of administration, from 0.09 to 0.15 μg intravenously or intramuscularly, up to 70 μg orally [1,4]. These differences highlight the gut’s role as a major barrier limiting systemic absorption of the toxin. The disease then progresses to a symmetric, descending flaccid paralysis affecting limbs and respiratory muscles up to respiratory failure. In certain situations, *C. botulinum* can bypass bacterial resistance to colonization, particularly in infants under one year of age, allowing bacterial growth and toxin production in the gut [5,6]. Infant botulism is the most frequently reported type of botulism in the United States each year, while in Europe, cases of foodborne botulism are mainly detected [2,7]. Although human botulism mortality rates have been reported to reach up to 60% before 1950 [7,8], advances in supportive care, including respiratory assistance, antibiotics, and access to serotherapy, have led to a substantial decline in mortality below 3% [9]. Nevertheless, the duration of hospitalization and length of stay in intensive care units (ICUs) continue to present a major burden to the healthcare system. Whereas BoNTs rank among the most potent biological protein toxins, when administered locally at appropriate doses, they serve as highly effective treatments for a variety of neuromuscular and glandular disorders [10,11].

The BoNT family is produced by *Clostridium botulinum* and related toxigenic anaerobic spore-forming *Clostridium* species, including *C. butyricum*, *C. baratii*, *C. novyi sensu lato*, *C. sporogenes* and *C. argentinense* [12]. Botulinum neurotoxins (BoNTs) are classified into seven major serotypes (A–G), which share 37–70% pairwise amino acid identity, and collectively encompass at least 40 subtypes that have significant differences in their primary amino acid sequences [13,14]. Serotype classification reflects marked antigenic differences, resulting in the inability of antisera generated against a given toxin to neutralize toxins belonging to a distinct serotype. Reported human foodborne botulism outbreaks are caused by BoNTs from serotypes A, B, E and F, while BoNTs of the serotypes C and D primarily cause disease in domestic and wild animals [2,14]. Adding to BoNT diversity is the growing number of reports describing BoNT-like toxins in *C. botulinum* and other bacterial genera, uncovered thanks to advances in large-scale genome sequencing, and whose host target species, including insects, largely remain to be identified [15,16,17,18]. For example, the BoNT-like toxin BoNT/X, which shows around 30% protein sequence identity with other BoNT serotypes, displays high enzymatic activity in vitro but low toxicity on human-induced pluripotent stem cell-derived neuronal cells and mice [17,19]. PMP1, a BoNT-like toxin targeting the malaria vector *Anopheles* mosquitoes, was identified in a mosquitocidal strain isolated from mangrove soil. [15]. A thorough understanding of the general mechanism of action of the botulinum neurotoxin family, as well as their distinct structural and functional properties, is essential for their rational exploitation as biotechnological tools.

**Figure 1:**
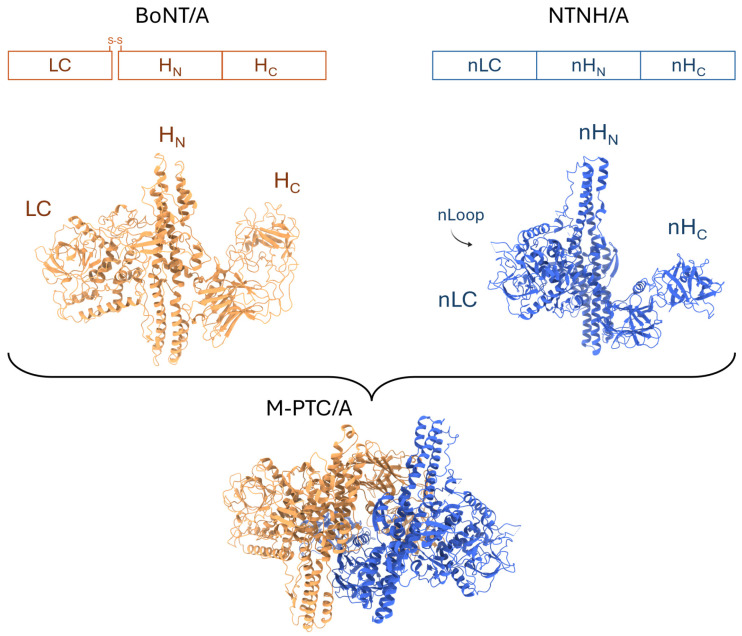
Three-domain architecture of BoNT/A and NTNH/A. Schematic representation of the BoNT/A di-chain and NTNH/A polypeptides, the three-domain architecture of BoNT/A and NTNH/A, as well as the structure of the minimal-progenitor complex (M-PTC/A). BoNT/A and NTNH/A share a similar overall fold. NTNH encoded in *ha* gene clusters contains a disorganized nLoop in LC that becomes structured upon interaction with the central pore-like channel of the HA70 trimer. BoNT/A and NTNH/A engage in handshake-like interactions to form the protective M-PTC/A complex. Note that the light chain (LC) of BoNT/A does not interact with NTNH/A. The protein ribbons shown in the figure correspond to M-PTC/A-VHH-F12 (3V0A) and separated BoNT/A and NTNH/A components.

BoNTs share a conserved structural organization into functional domains (Figure 1) [20,21]. Each BoNT is synthesized as a single-chain polypeptide of around 150 kDa, which must be proteolytically processed into a di-chain molecule composed of an enzymatically active light chain (LC, ~50 kDa) and a heavy chain (HC, ~100 kDa), linked together by a disulfide bond (Figure 1) [22]. The LC functions as a zinc-dependent metalloprotease that cleaves soluble *N*-ethylmaleimide–sensitive factor attachment receptor (SNARE) proteins at cholinergic neuron endings, thereby blocking synaptic vesicle fusion required for neurotransmitter release and muscle contraction [21]. The HC is primarily subdivided into two functional domains comprising a N-terminal translocation domain (H_N_), forming long α-helices, which mediates delivery of the LC across endosomal membranes into the cytosol, and the C-terminal binding domain (H_C_), which recognizes complex gangliosides together with specific protein receptors enriched at neuron endings [20,23]. These polysialogangliosides, comprising a ceramide tail and a carbohydrate head group displaying various numbers of sialic acids attached, are particularly enriched at the presynaptic membrane of neurons, a key determinant in the targeting of motoneuron endings by neurotoxins [24]. Although the three functional domains are typically distinct, H_N_ forms an unusual belt that wraps around the catalytic domain [20].

**Figure 2:**
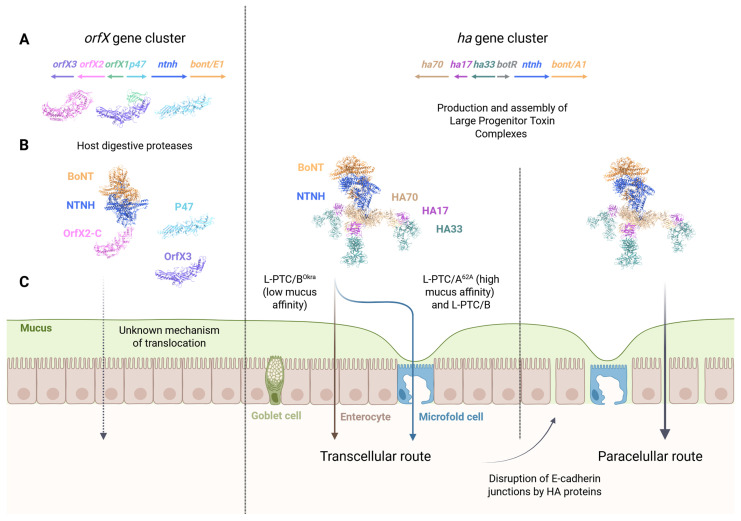
Assembly and absorption pathways of botulinum neurotoxin complexes. (**A**) Two types of BoNT-encoding gene clusters can be distinguished depending on the presence of genes encoding either OrfXs/P47 (left panel) or HA (right panel) NAPs along with a gene encoding nontoxic non-hemagglutinin protein (NTNH). (**B**) BoNTs form interlocked complexes (M-PTCs) with NTNHs at acidic pH, protecting each other from a harsh proteolytic environment. Left panel: NTNH associates with OrfX2, after proteolysis of its N-terminal tubular lipid-binding (TULIP) domain (OrfX2-N), freeing the carboxy-terminal part of OrfX2 (OrfX2-C). Note that OrfX1 is also proteolyzed by digestive proteases, freeing the carboxy-terminal part of OrfX3. Right panel: HA proteins associate with NTNHs to form large hetero-dodecameric progenitor toxin complexes (L-PTCs). (**C**) In contrast to HA-type L-PTCs (right panel), the absorption pathway of OrfX-type BoNT remains to be characterized (left panel). Depending on the capacity of L-PTCs to interact with fucosylated mucins, L-PTCs first undergo transcytosis through enterocytes (L-PTC/B^Okra^) or M cells (L-PTC/A^62A^), which are covered by a thin mucus layer. Following transcytosis, HA-induced disruption of intercellular junctions facilitates the absorption of the luminal fraction of the toxin, enabling optimal systemic dissemination. The protein ribbons displayed in the figure correspond to full-length OrfX2 (PDB 8FBF), M-PTC-OrfX2-C/E (PDB 9ARJ), OrfX1:OrfX3/E (PDB 8FBD), P47/E (PDB 5WIX) and L-PTC/B (PDB 9QCM).

Each BoNT is encoded within large gene clusters together with a nontoxic non-hemagglutinin (NTNH) protein and a set of other neurotoxin-associated proteins (NAPs) (Figure 1 and Figure 2A). BoNTs and NTNHs are co-produced with three classes of hemagglutinins (HAs) or four types of OrfXs/P47 NAPs. For example, BoNT/A1, A5, B, C, CD, DC, D, or G are encoded within *ha* gene clusters, while BoNT A1–A4, A6–A8, E, F, and H belong to *orfX*-type gene clusters, as for recently described BoNT-like toxins [15,25,26,27]. In rare instances, BoNTs, such as the A1 subtype, may be associated with either *ha*- or *orfX*-gene clusters, depending on the strain. The NTNH component binds directly to BoNT, forming the minimal-progenitor toxin complex (M-PTC) (Figure 1) [28,29,30,31]. Owing to structural similarities with BoNTs, NTNHs can be subdivided into domains referred to as nLC, nH_N_, and nH_C,_ bearing in mind that these domains do not display the molecular features that ensure BoNT functions (Figure 1). The NTNH instead contributes to burying an unusually large solvent-accessible area of BoNT through multivalent interfaces. At the center of the complex lies the HC fragment, which is surrounded by all three domains of NTNH (Figure 1). For major serotypes, this interlocked handshake structure of M-PTCs that forms at acidic pH is disrupted when the pH becomes neutral, which is thought to occur after M-PTCs complete transcytosis across the intestinal epithelium, thereby allowing BoNTs to be released in the circulation [31,32]. Recent findings highlight the importance of repulsive forces at neutral pH in promoting the dissociation of BoNTs from NTNHs [31]. Finally, NTNHs not only protect BoNTs but also serve as a molecular platform for interactions with NAPs, as reviewed here (Figure 2B). Both NTNHs and NAPs convergently address the pathophysiologic needs of BoNTs to overcome proteolytic degradation in the stomach and enhance neurotoxin translocation across the intestinal barrier, both by transcellular and paracellular pathways (Figure 2C). This rate-limiting step of crossing the intestinal epithelial barrier is thought to contribute to host species specificity and intoxication efficacy according to BoNT subtypes.

In this review, we aim to synthesize recent structural and functional insights into how NTNHs form an assembly platform that links BoNTs to HAs or the mature form of OrfX2 (OrfX2-C), highlighting the role of NAPs in oral toxicity. We further discuss how the carbohydrate binding specificity of large-progenitor toxin complexes (L-PTCs) dictates the route of intestinal absorption, followed by E-cadherin targeting for cell–cell junction disruption, which enables paracellular translocation of BoNTs for full systemic dissemination.

## 2. Mode of Interaction of NTNH with the Mature Form of OrfX2

The work of *Gao* et al. establishes a first direct association between OrfX-type NTNH and one of the OrfXs/P47 components, providing a demonstration of the key role of this interaction in promoting efficient oral intoxication of mice by BoNT/E1 and OrfX-type BoNT/A1 from strain ST7B [33] (Figure 1). This extends previous findings showing that NTNH and OrfX1-3 from *B. thuringiensis* strain 4Q7 enhance the toxicity of the BoNT-like toxin PMP1 on the major malaria vector *Anopheles coluzzi* [15,16]. More recent work used CRISPR–Cas9 gene editing in the *C. botulinum* E1 Beluga strain and employed mouse models of intoxication via oral gavage versus intraperitoneal injection, enabling assessment of BoNT lethal activity independently of rate-limiting intestinal absorption [33]. By recording the median lethal dose (LD_50_) in both settings, this work provides compelling evidence that the OrfXs/P47 NAPs are required to enhance approximately 50-fold the toxicity of M-PTC/E1 when given by the oral route, while being devoid of intrinsic toxicity [33]. This represents the first demonstration of a specific role of OrfXs/P47 in oral intoxication of vertebrates, likely through promoting efficient absorption of BoNT/E1 from the gut to the circulation. As discussed below, these findings were extended to provide molecular evidence connecting the M-PTC/E1 to a protein of the OrfXs/P47 complex once it has been matured by host digestive proteases, thereby resolving a long-standing question in the field about the possible interplay between OrfXs/47 and M-PTC [33].

The OrfXs/P47 components exhibit notable structural similarities to one another and to tubular lipid-binding (TULIP) domains (Figure 2) [34,35,36,37]. TULIP fold primarily consists of a long α-helix that is wrapped by a twisted β-sheet composed of anti-parallel β-strands [37,38]. These TULIP domains serve as a structural scaffold forming a hydrophobic cavity for the binding of large lipids or hydrophobic molecules [37]. P47 adopts a fold topology that displays similarity to Bactericidal Permeability Increasing Protein (BPI), as well as Lipopolysaccharide binding protein (LBP) and cholesteryl ester transfer protein (CETP), despite low primary sequence identity [34,35,38]. BPI is composed of a central seven-stranded beta-sheet flanked by two TULIP-like domains that are connected by a loop [38]. Indeed, the carboxy-terminal domain of P47 displays a structural homology with the amino-terminal TULIP domain of BPI, while its amino-terminal part has a more compact TULIP-like structure that does not superimpose well with BPI. The structure of OrfX3 shows similarities to that of P47 [34,36]. OrfX2 exhibits a comparable topology to OrfX3, except that it contains an extra N-terminal TULIP domain displaying structural homology with OrfX1 [36,39]. The N-terminal TULIP-like domain of OrfX3 is able to associate with OrfX1 [34,36]. Thus, the overall architecture of OrfX2 resembles that of the OrfX1:OrfX3 complex (Figure 2A) [36]. Together, this establishes that OrfXs/P47 belong to the TULIP superfamily and suggests that OrfXs/P47 may associate with lipids [34,35]. Consistent with this idea, P47 induced the aggregation of liposomes composed of a mixture of different phospholipids and cholesterol [35].

Until recently, molecular and functional links connecting OrfXs/P47 factors to M-PTC were missing. Recent work demonstrates that oral toxicity triggered by BoNT/E1 involves interactions between NTNH/E1 and a mature form of OrfX2, e.g., once the N-terminal TULIP-like domain (residues 1–164) of OrfX2 has been proteolytically hydrolyzed by host digestive proteases into an OrfX2-C polypeptide [33]. In addition, the authors show that pepsin digests OrfX1 within the OrfX1–OrfX3 complex, thereby freeing OrfX3 for an unknown function. Remarkably, biochemical analyses show that pepsin digestion or recombinant production of OrfX2-C exposes an N-terminal surface of OrfX2-C that becomes able to bind to NTNH/E1, either alone or once engaged in interactions with BoNT/E1 (M-PTC/E1). Of note, the addition of recombinantly produced OrfX2-N to OrfX2-C competes for the binding of OrfX2-C to NTNH/E1, thereby demonstrating the capping function of OrfX2-N.

Cryo-electron microscopy (cryo-EM) data reveal that the surface exposed at the N-terminal domain of OrfX2-C associates with NTNH/E1 at two non-overlapping major and minor occupancy sites. Occupancy refers to the percentage of each surface in NTNH/E1 engaged in interactions with the N-terminal surface of OrfX2-C, as visualized by cryo-EM. Moreover, both binding sites of OrfX2-C in NTNH/E1 can be occupied by one OrfX2-C molecule at the same time, even though it is less frequently observed. The major binding interface of NTNH/E1 involves four loops within the nLC domain, while the minor binding interface involves elements borrowed from both nH_N_ and nH_C_ domains of NTNH. The introduction of point mutations that specifically disrupt interactions at the minor and major binding sites between NTNH/E1 and OrfX2-C convincingly established that the major site plays a critical role in enhancing oral intoxication by BoNT/E1, while also indicating the involvement of the minor site [33]. Finally, these data are further broadened by demonstrating the interchangeability of OrfXs/P47 between the BoNT/E1-producing strain Beluga and the OrfX-type BoNT/A1-producing strain ST7B [33].

These findings, pointing to a role for OrfXs/p47 at the interface of small intestine epithelium, may provide broader insights into the mechanisms of bacterial virulence, as many *orfX* gene clusters are found in BoNT non-producing bacteria from diverse phylogenetic backgrounds [25]. Indeed, *orfX* gene clusters are located near genes related to insecticidal toxins or toxic protein domains. In conclusion, this comprehensive work lays the foundation for future studies on how OrfXs/P47 complexes enhance toxin action at the interface of the intestinal barrier to promote oral intoxication, a mechanism certainly borrowed by BoNTs through evolution.

## 3. Mode of Interaction of NTNH with HA-Type Complex

In parallel, progress has been made in defining how HA components assemble with M-PTC to form a large progenitor toxin complex (L-PTC) [30,31]. Recent findings shed new light on the molecular mechanism by which M-PTC is anchored at the center of the three-arm hetero-dodecameric HA70:HA17:HA33 (stoichiometry 3:3:6) complex [40,41,42] (Figure 2B). Indeed, previous studies had established that the trimer of HA70 forms a symmetrical triskelion-like platform displaying three extensions each formed by HA17:HA33 (stoichiometry 1:2) molecules, and, at its center, a less defined interaction with HA-type NTNH involving a flexible nLoop located in the nLC domain (Figure 1) [40,41,42,43]. This nLoop, which is not found in OrfX-type NTNH, turned out to play key roles in the association of HA-type M-PTC with the HA70 trimer.

In the triskelion platform, three HA70 monomers contribute four antiparallel β-strands each, forming a central pore with a negatively charged inner surface similar to that of the aerolysin toxin from *Aeromonas hydrophila*. Recent studies provide high-resolution cryo-EM structures of M-PTC/A1 and B1 in complex with the HA70 trimer [30,31]. As shown for NTNH/B1, the core of the nLoop in the nLC domain of NTNH/A1 (amino acids Met122–Pro144) undergoes a transition from a disordered segment to a β-hairpin, containing two short antiparallel β-strands that dock at the entrance of the pore formed by the HA70 trimer (Figure 1) [30,31]. The tip of the nLoop twists within the central opening of the pore as it forms extensive contacts. Thus, the negatively charged surface of the pore becomes engaged in extensive interactions with a positively charged amino acid motif, KSNKK, at the tip of the nLoop (amino acids Gly111–Ala149 in NTNH/B1). In addition, this work suggests that HA70 monomers assemble cooperatively around the nLoop, rather than the nLoop docking into a preformed pore [31].

The nLoop inserted into the pore constitutes a major interaction interface between NTNH and the HA70 trimer. This accounts for roughly one-third of the total interface [31]. The interaction is further stabilized by additional hydrophobic interactions between three loops in the nLC domain and three loops of the HA70 triskelion-like platform [30]. In the M-PTC/B1 complex, although specific hydrophobic interactions are observed between the HC domain of BoNT/B1 and the HA70 trimer, their disruption has no detectable impact on the formation of the complex [31].

Interaction of the nLoop of NTNH/A1 with the HA70 trimer only tolerates single mutations in the nLoop (M122K, I143K, or F145H) [30]. Together with the conservation of nLoop amino acid sequences between BoNT serotypes (22 conserved amino acids out of the 33 forming the nLoop), this suggests interchangeability between nLoops and HA70 produced in different HA-type gene clusters [31]. Consistently, NTNH/A1 can associate with trimers of HA70 from serotypes A, B, D, and G [30].

In conclusion, two complementary studies provide a comprehensive view of NTNH: HA70 trimer assembly, highlighting the central role of the nLoop that is found in NTNH produced in *ha* gene clusters specifically [30,31].

## 4. The Intestinal Barrier and Neurotoxin Absorption

Recent work on decrypting the molecular mechanism of L-PTC assembly parallels the extensive progress made in deciphering the HA-dependent mode of interaction of L-PTCs with the intestinal epithelium for transcytosis and disruption of the epithelium barrier [44,45,46,47,48]. Note that BoNT/A and B have an intrinsic capacity to associate with intestinal epithelia, in a ganglioside-dependent manner, for transcytosis, albeit with lower efficacy as compared to M-PTC or L-PTC [45,49,50]. The L-PTC of serotype B exhibits approximately 700-fold higher oral toxicity than M-PTC/B, which is about 20-fold more toxic than BoNT/B [51,52]. The intestinal barrier is a composite structure consisting of a single epithelial cell layer covered by a protective mucus layer [53]. The dynamic epithelial interface mediates selective permeability through several specialized epithelial cell types. Enterocytes, equipped with dense microvilli that form the absorptive brush border, represent 80% of all epithelial cells in the small intestine. Other cell types include Goblet cells that secrete mucins involved in the formation of the mucus hydrogel, which protects the epithelium from mechanical injury, microbial invasion, and digestive enzymes. Microfold (M) cells, found primarily in the follicle-associated epithelium of Peyer’s patches and other gut-associated lymphoid tissues, facilitate antigen sampling. Epithelial cells express E-cadherin, which forms adherens junctions that ensure cell–cell cohesion and tight junctions’ integrity to prevent diffusion of molecules through the epithelium barrier. It is worth noting that a disruption of the epithelial barrier can be directly triggered by the C2 toxin or C3 exoenzyme, produced by certain strains of *C. botulinum* from serotypes C and D [54,55]. These toxins target the actin cytoskeleton either directly or indirectly through inhibition of the small GTPase RhoA [54,55]. Nevertheless, in most cases, the absorption of L-PTCs across the intestinal barrier is achieved through their binding (1) to specific carbohydrates for transcytosis [47,56], and (2) to E-cadherin, after completion of transcytosis for a disruption of cell–cell junctions and opening of a paracellular route (Figure 2C) [32,57,58]. As discussed below, we are beginning to appreciate the molecular determinants that govern L-PTC translocation efficiency. Critical steps involved in crossing the intestinal barrier vary markedly between host species and BoNT subtypes, accounting for differences in intoxication efficiency and thus host specificity.

Extensive protein structure determination, coupled with biochemical analysis of hemagglutinins from *C. botulinum* strain 62A, had already shed light on their mode of interaction with glycoconjugates [40]. There is no overlap in carbohydrate-binding selectivity between HA70 and HA33 [40]. HA33 adopts a dumbbell-shaped structure composed of two beta-trefoil domains (Ricin B-like lectin superfamily) linked together by an alpha-helix [40,59]. The carboxy-terminal part of HA33 from serotypes A and B binds to galactose decorating the terminal part of glycans [40,60]. Unlike HA33, each HA70 molecule binds glycans terminating in *N*-acetylneuraminic acid (Neu5Ac), the common sialic acid, primarily through an extended flat surface, forming six hydrogen-bond pairs [40]. This likely confers flexibility for additional glycan binding beyond the terminal Neu5Ac. Of note, HA33 from serotypes C and D has two carbohydrate-binding sites for association with galactose and Neu5Ac with high affinity, while each H70/A-D has a conserved sialic acid-binding site, which therefore probably does not contribute to defining the specificity of host intoxication [40,61]. In line with this, previous studies have established that HA33/C can affect host epithelial cell viability through sialic-acid-dependent binding to GM3 gangliosides [44,62].

New work points to carbohydrate binding and L-PTC entrapment in the *O*-glycosylated mucin layer of the mucus as key determinants in dictating the route and efficiency of L-PTC absorption from the intestine [48]. Earlier studies established that HA-type L-PTCs can be classified as either hyper–oral toxic (HOT) or non-HOT toxins, regardless of the serotype, and despite similar parenteral toxicities [48,63]. Recent work establishes that fucosylation of the terminal galactose in *O*-glycosylated mucin accounts for a 20–80-fold higher oral toxicity of L-PTC/B1 strain Okra, as compared to L-PTC/A1 strain 62A [48]. Fucose is a 6-desoxygalactose that is incorporated into *N*-linked glycans, exposed at the surface of epithelial cells, as well as incorporated into *O*-type glycans that decorate mucins. The L-PTC/B-Okra largely avoids binding of the α1,2-fucosylated terminal galactose of mucins and therefore enters enterocytes within the villus epithelium, which represents a large surface of absorption. In contrast, L-PTC/A-62A binds the α1,2-fucosylated terminal galactose of mucins and is trapped in the gel-forming mucus layer. Consistently, the fraction of L-PTC/A-62A that escapes entrapment is preferentially taken up by microfold (M) cells, above which the mucus layer is thinner. This differential interaction of L-PTCs with epithelial cells is primarily dictated by differences in the carbohydrate-binding pocket of HA33 [48]. Structure modeling coupled to biochemical analyses of the galactose binding pocket shows the importance of the residue H281 in HA33/A-62A, as compared to N282 in HA33/B-Okra, which does not accommodate the α1,2-fucosylated form of galactose [48]. Thus, HA33/B-Okra had a higher affinity for mouse intestinal mucin prepared from mice lacking fucosyltransferase 2, which exposes the underlying galactose residues, compared with mucin prepared from wild-type animals [48]. In line with this, the oral toxicity of L-PTC/B-Okra is significantly reduced in mice lacking fucosyltransferase 2, the enzyme responsible for α1,2-fucosylation of proteins and lipids throughout the gastrointestinal tract. Note that variations in amino acid residues within the carbohydrate-binding pocket of HA33, which determine its affinity for the α1,2-fucosylated form of galactose, are not associated with the BoNT serotypes and may contribute to predicting the oral toxic potential among L-PTCs [48]. In good agreement with these findings, L-PTC/A-62A exhibits comparable toxicity in both animal models. Together, these novel findings extend previous data showing that the oral toxicity of L-PTC/A-62A is dramatically reduced in M-cell-depleted mice and also point to the importance of glycoprotein 2 (GP2) as a receptor in M cells [56]. Therefore, HA molecules dictate the route of transcytosis through specific intestinal epithelial cells, depending on their spectrum of association to carbohydrates with the involvement of *O*-glycosylated mucins as key players [48]. These HA proteins also play a key role in disrupting monolayer cohesion to promote neurotoxin absorption, as discussed below.

HA complexes L-PTC/A-62A and L-PTC/B-Okra can disrupt epithelial tight junctions following transcytosis to the basolateral side of the epithelial monolayer, through direct binding to E-cadherin in a glycan-independent manner [47,57,58]. Nevertheless, the carbohydrate-binding activities of HAs facilitate barrier disruption triggered by L-PTC, likely favoring HAs binding to the membrane for interactions with E-cadherin [60]. The epithelial barrier-disrupting activity of L-PTC promotes paracellular transport of BoNT, thereby enhancing oral toxicity, after the toxin has transcytosed through enterocytes (L-PTC/B) or M cells (L-PTC/A) [47] (Figure 2C). The interaction between HA proteins and mouse, human, or bovine E-cadherin is absent in toxin-resistant rat and chicken species due to the presence of an arginine residue at position 20 in the E-cadherin sequence [60]. HA proteins from BoNT serotype C do not bind human E-cadherin, consistent with epidemiological evidence showing that BoNT/C is primarily responsible for animal botulism, despite a susceptibility of human neurons to this serotype [44,60]. Indeed, botulism type C is mainly found in animals (birds, cattle), and rarely in human botulism, while type A is responsible for severe forms of human botulism. Crosstalk between HA proteins and E-cadherin is most likely an important determinant of host susceptibility to BoNT following oral challenge [32,57,58].

In conclusion, the host-specific epithelial barrier targeting and disruption, as well as carbohydrate-binding properties of HA proteins between L-PTCs, contribute to shaping distinct host susceptibility through intrinsic capacities to modulate intestinal absorption of the neurotoxins.

## 5. Conclusions and Perspectives

Recent advances provide a detailed mechanistic understanding of how NTNH coordinates with OrfX2-C or HA complex to promote BoNT absorption across the intestinal barrier. The refined structure of the nLoop of NTNH, anchored in HA70 triskelion-like element and protease-dependent activation of OrfX2 for association to NTNH, underscores the complexity and adaptability of BoNT assembly strategies. These findings pave the way for translational research, such as designing targeted inhibitors to improve countermeasures against botulism. Future studies should focus on the dynamics of BoNT transcytosis and trafficking from gut to neurons, including the interplay between host factors and progenitor complexes, which will be critical for mitigating the public health impact of such potent toxins.

## Data Availability

No new data were created or analyzed in this study.

## Abbreviations

The following abbreviations are used in this manuscript:

BoNT	Botulinum neurotoxins
HA	Hemagglutinin
L-PTC	Large-Progenitor toxin complex
M-PTC	Minimal-Progenitor toxin complex
NAP	Neurotoxin-associated proteins
NTNH	Nontoxic non-hemagglutinin factor
TULIP	Tubular lipid-binding
